# Diagnostic yield of endoscopy in women under 50 with iron deficiency anemia: a retrospective cohort study from Jordan

**DOI:** 10.3389/fgwh.2026.1805913

**Published:** 2026-07-14

**Authors:** Awni Abu Sneineh, Sara Haj Ali, Bashar Quteishat, Neebal Musleh, Dana Aldabas, Mai Awad, Suleiman Alatiyat, Rahaf Salah, Laith Deeb, Tala Ghunim, Leen Albassoumi, Nada Khraisat, Rasha Ali

**Affiliations:** 1 Department of Internal Medicine, Faculty of Medicine, University of Jordan, Amman, Jordan; 2 Department of Internal Medicine, Faculty of Medicine, Al-Balqa Applied University, Salt, Jordan

**Keywords:** colonoscopy, endoscopy, gastrointestinal symptoms, iron deficiency anemia, premenopausal

## Abstract

**Background and aims:**

Iron deficiency anemia is a serious health issue that is largely prevalent, especially in premenopausal women. Guidelines recommend endoscopic evaluation of these patients even in the absence of symptoms. However, the evidence for this recommendation is weaker than that for postmenopausal women. Our study aimed to determine the diagnostic yield of endoscopic evaluation of women under 50 years with iron deficiency anemia.

**Methods:**

We performed a dual-center retrospective cohort study over a period of three years. Consecutive females aged 18–49 years with Hb <12 g/dL and ferritin <45 ng/mL who underwent upper and/or lower endoscopy for iron deficiency anemia were included.

**Results:**

We included 300 women [median age 39.0 years (Interquartile range 29.0–45.0) and median Hb of 10.7 g/dL (Interquartile range: 9.3–11.6)]. The vast majority (82.7%) reported gastrointestinal symptoms. Upper endoscopy revealed abnormalities in 79.4% of patients, most commonly gastritis (57.0%). Colonoscopy showed abnormalities in 48.6%, primarily internal hemorrhoids (32.6%). Significant findings were identified in 19.3% of patients, with older age being the only significant predictor. The prevalence of specific diagnoses was 2.0% for celiac disease, 1.7% for inflammatory bowel disease, and 1.3% for colorectal cancer. No clinical variables predicted these specific diagnoses.

**Conclusion:**

Endoscopic evaluation of young women with iron deficiency anemia identifies a high burden of gastrointestinal pathology, predominantly in the upper tract. The inability of symptoms or other clinical features to predict significant findings supports a broad referral strategy for endoscopic investigation in this population.

## Introduction

Iron deficiency anemia (IDA) is the most prevalent type of anemia. According to the World Health Organization, 30% of women of childbearing age worldwide are anemic, with IDA contributing to at least half of the cases (1). In Jordan, it was found that 28.7% of women had iron deficiency, with crude prevalence of anemia of 20.5% (2).

Causes of IDA include inadequate iron intake, malabsorption, or blood loss. In men and postmenopausal women, blood loss is usually through the gastrointestinal (GI) tract, while in premenopausal women, it is mostly due to obstetric/gynecological causes (3). Causes of IDA that originate from the GI tract include celiac disease, inflammatory bowel disease (IBD), and peptic ulcer disease, as well as malignancy, for which the reported prevalence in patients with IDA can be as high as 12% (4).

The diagnosis of IDA relies on testing iron profile with a low serum ferritin level being the most commonly used for confirming iron deficiency. The American Gastroenterological Association (AGA) recommends using a ferritin cutoff value of 45 ng/mL to diagnose iron deficiency (5) which yields higher sensitivity (85%) and acceptable specificity (92%) compared to the traditional 15 ng/mL cutoff, which has 59% sensitivity and 99% specificity (6).

The AGA strongly recommends endoscopic evaluation for men and postmenopausal women with IDA who lack GI symptoms and have no explanation for their anemia. For premenopausal women with IDA and no obvious cause, the AGA suggests endoscopic evaluation rather than solely relying on iron replacement therapy. For IDA patients who have GI symptoms, the AGA recommends an endoscopic workup guided by symptoms in both groups (5). If the endoscopic evaluation does not find a cause for the anemia, the AGA recommends testing for Helicobacter pylori (h. pylori) infection non-invasively and treating it if present because of the link between h. pylori and IDA (7). In contrast, the British Society of Gastroenterology do not recommend endoscopic workup of premenopausal women with IDA unless they were older than 50 years, had alarming symptoms, had family history of colorectal cancer (CRC) or had undergone hysterectomy (8). As there are no randomized studies comparing endoscopic evaluation to conservative treatment with iron replacement in premenopausal women, these guidelines were based on the low prevalence of CRC in patients under fifty years (9) and several observational studies on the diagnostic yield of upper and lower endoscopy in young patients. The majority of these studies included mixed cohorts, often combining symptomatic and asymptomatic patients, as well as males and females. Moreover, data specifically addressing premenopausal women in Jordan and the Middle East are lacking.

Our study primarily aimed to determine the proportion of abnormal endoscopic findings in Jordanian women under 50 years with IDA. Additionally, we aimed to characterize the specific GI lesions identified during endoscopic examinations of these patients and to investigate whether the detection of a lesion on endoscopy was associated with the patient's age, severity of anemia or presence of GI symptoms.

## Methods

We conducted a retrospective descriptive observational cohort study at two tertiary referral hospitals in Jordan, serving two governorates (equivalent to provinces) in the central region: Al-Hussein Salt Hospital (a public hospital) and Jordan University Hospital (a teaching hospital). Electronic medical records from the endoscopy unit databases of both hospitals were retrospectively reviewed for consecutive cases of women aged 18–49 years with IDA who underwent upper and/or lower endoscopy in the period between June 2022 and June 2025. Patients were excluded if they had known diagnosis of IBD, celiac disease, or a history of GI malignancy, or if their procedure was incomplete, preventing a full evaluation. Data collected included age, hemoglobin (Hb) and ferritin levels, upper and/or lower endoscopic findings, histopathology results if biopsy was taken, results of H. pylori testing (stool antigen, urease test or histopathology), history of menorrhagia (if documented), and celiac serology. Due to the retrospective nature of the study, a standardized approach to quantify menstrual blood loss was not feasible. Therefore, we defined a case as positive for menorrhagia based on any medical record documentation of the term ‘menorrhagia'.

Abnormal upper endoscopy findings were defined as reflux esophagitis, varices, hiatus hernia, gastritis (based on biopsy showing H. pylori or chronic active gastritis), duodenitis, peptic ulcer, arteriovenous malformation, polyps or masses. Abnormal colonoscopy findings were defined as internal hemorrhoids, solitary rectal ulcer, colitis, ileitis, arteriovenous malformation, diverticulosis, polyps or masses. A significant endoscopic finding was defined as polyp/mass, ulcer, arteriovenous malformation, portal hypertension, endoscopic appearance suggestive of celiac disease (scalloping, fissuring or loss of duodenal folds) or findings suggestive of IBD. Diagnosis of celiac disease required both histologic findings consistent with Marsh class 2 or above and a positive anti-tissue transglutaminase IgA antibody. Diagnosis of IBD was based on compatible endoscopic features (loss of vascular markings, mucosal ulceration, friability or pseudopolyps) and a histologic confirmation (crypt architectural distortion, branching, or atrophy, and lamina propria mononuclear cell infiltration).

For this descriptive study, the sample size was calculated for precision of estimation. Using Cochran's formula for a single proportion, a minimum sample of 289 patients was needed to estimate a diagnostic yield of 25% with a 5% margin of error and a 95% confidence level. We planned to include all eligible patients during the study period, even if this number exceeded the minimum required sample size, as this would improve the precision of the estimates.

Ethical approval was obtained from the institutional review board at Jordan University Hospital (approval number 10/2025/25650) and the institutional review board at Al-Balqa Applied University (approval number 11391). Both IRBs waived the requirement for informed consent given the retrospective nature of the analysis. The study was conducted in accordance with the Declaration of Helsinki.

### Statistical analysis

Statistical analyses were performed using SPSS version 21. Continuous variables were tested for normality using the Shapiro–Wilk test and as they did not follow a normal distribution, they were presented as median and interquartile range (IQR) and analyzed using the Mann–Whitney U test for comparisons between groups. Categorical variables were expressed as frequencies and percentages and analyzed using the Chi-square or Fisher's exact tests, depending on expected cell counts. For variables with missing data (H. pylori: 25.0%; menorrhagia: 55.7%), we performed complete-case analysis. Only patients with documented values were included in crosstabulations involving those variables. *P*-value <0.05 was considered statistically significant.

## Results

### Demographic and clinical characteristics of the study participants

**Figure 1:**
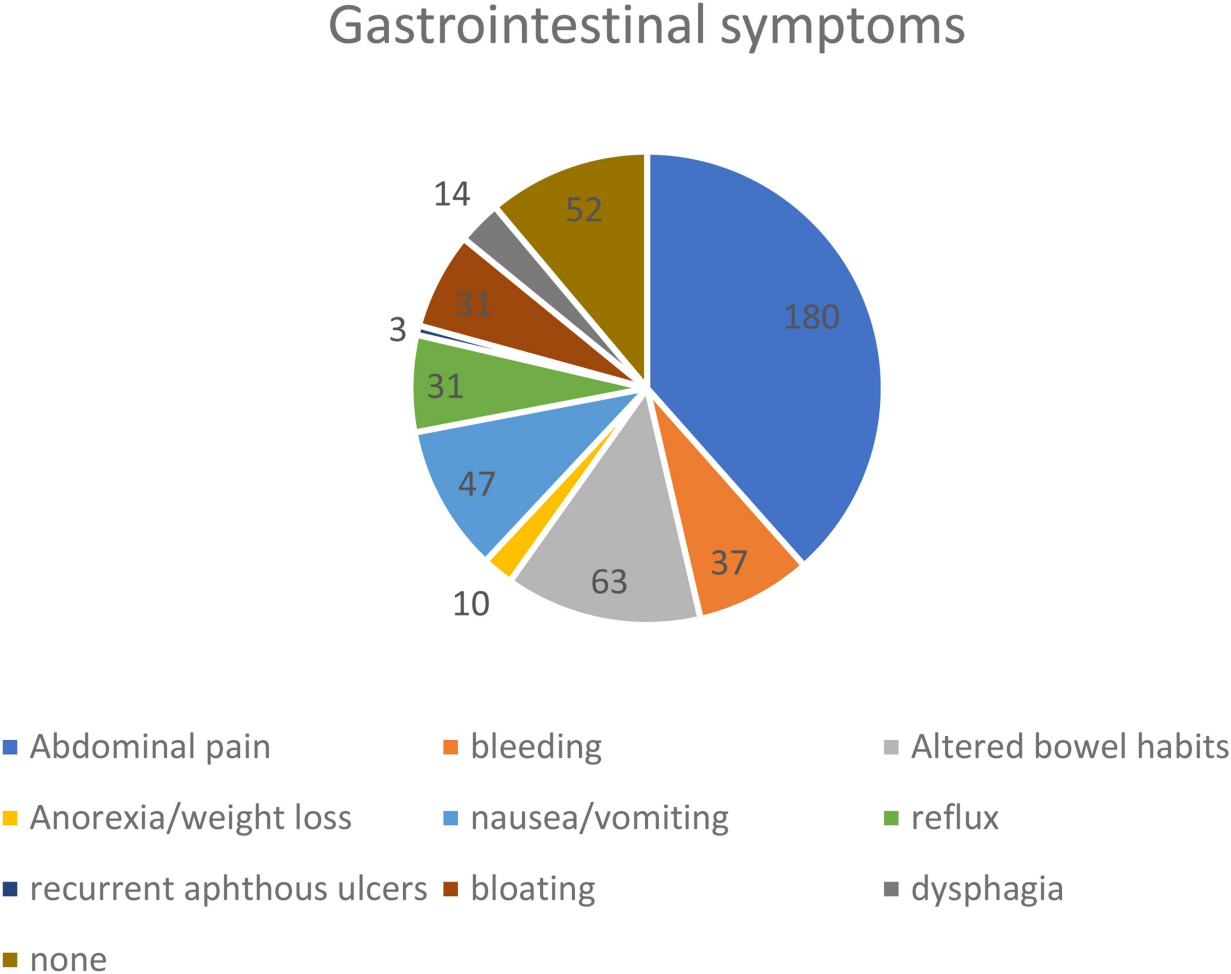
Frequency of gastrointestinal symptoms among the study cohort (*n* = 300). Gastrointestinal symptoms were recorded among women under 50 years of age with iron deficiency anemia at Jordan University Hospital and Al-Hussein Salt Hospital between June 2022 and June 2025. Some patients presented with more than one symptom.

Over the three-year period, 300 women fulfilled our criteria. The median age was 39.0 years (IQR 29.0–45.0), median hemoglobin level was 10.7 g/dL (IQR 9.3–11.6) and the median ferritin level was 5.9 ng/mL (IQR 2.9–10.8). Most participants (82.7%) reported GI symptoms, with abdominal pain being the most common Figure 1. Bidirectional endoscopy was performed in 163 patients (54.3%). The remaining 137 patients had a single procedure depending on their symptoms: 119 (39.7%) underwent upper endoscopy only and 18 (6%) underwent colonoscopy only. H. pylori status was documented for 225 patients (75.0%), of whom 121 (53.8%) were positive. A history of menorrhagia was documented for 133 patients (44.3%), and of these, 56 (42.1%) were reported to have menorrhagia. Overall, significant endoscopic findings were detected in 58 patients (19.3%). Patients with significant finding were significantly older than those without [median age 43.5 years [IQR 35.0–47.0] vs. 38.0 years [IQR 28.0–44.3], *p* = 0.011]. No other variables showed a statistically significant association. Table 1 compares the characteristics of patients with and without significant findings.

**Table 1:** Characteristics of the study participants.

Variable	Significant finding (*N* = 58)	No significant finding (*n* = 242)	*P* value
Age, years (median, IQR)	43.5 [IQR 35.0–47.0]	38.0 [IQR 28.0–44.3]	0.011^a^
Hb, g/dL (median, IQR)	10.7 [IQR 9.3–11.8]	10.6 [IQR 9.3–11.6]	0.884^a^
Ferritin, ng/mL (median, IQR)	7.7 [IQR 3.1–11.2]	5.7 [IQR 2.8–10.9]	0.339^a^
GI symptoms			0.452^b^
Yes	46 (79.3%)	202 (83.5%)	
No	12 (20.7%)	40 (16.5%)	
H. pylori status^c^			0.702^b^
Positive	22/43 (51.2%)	99/182 (54.4%)	
Negative	21/43 (48.8%)	83/182 (45.6%)	
Menorrhagia^d^			0.783^b^
Positive	15/34 (44.1%)	41/99 (41.4%)	
Negative	19/34 (55.9%)	58/99 (58.6)	

Baseline demographic and clinical characteristics of women under 50 years with iron deficiency anemia undergoing endoscopic evaluation at Jordan University Hospital and Al-Hussein Salt Hospital between June 2022 and June 2025.

IQR, interquartile range; Hb, hemoglobin; GI, gastrointestinal; H. pylori, Helicobacter pylori.

a Mann–Whitney U test.

b Chi square.

c H. pylori status was documented in 225 patients.

d Menstrual bleeding was documented in 133 patients.

*P* value significant if <0.05.

### Upper endoscopic findings in women under 50 years with iron deficiency anemia

**Figure 2:**
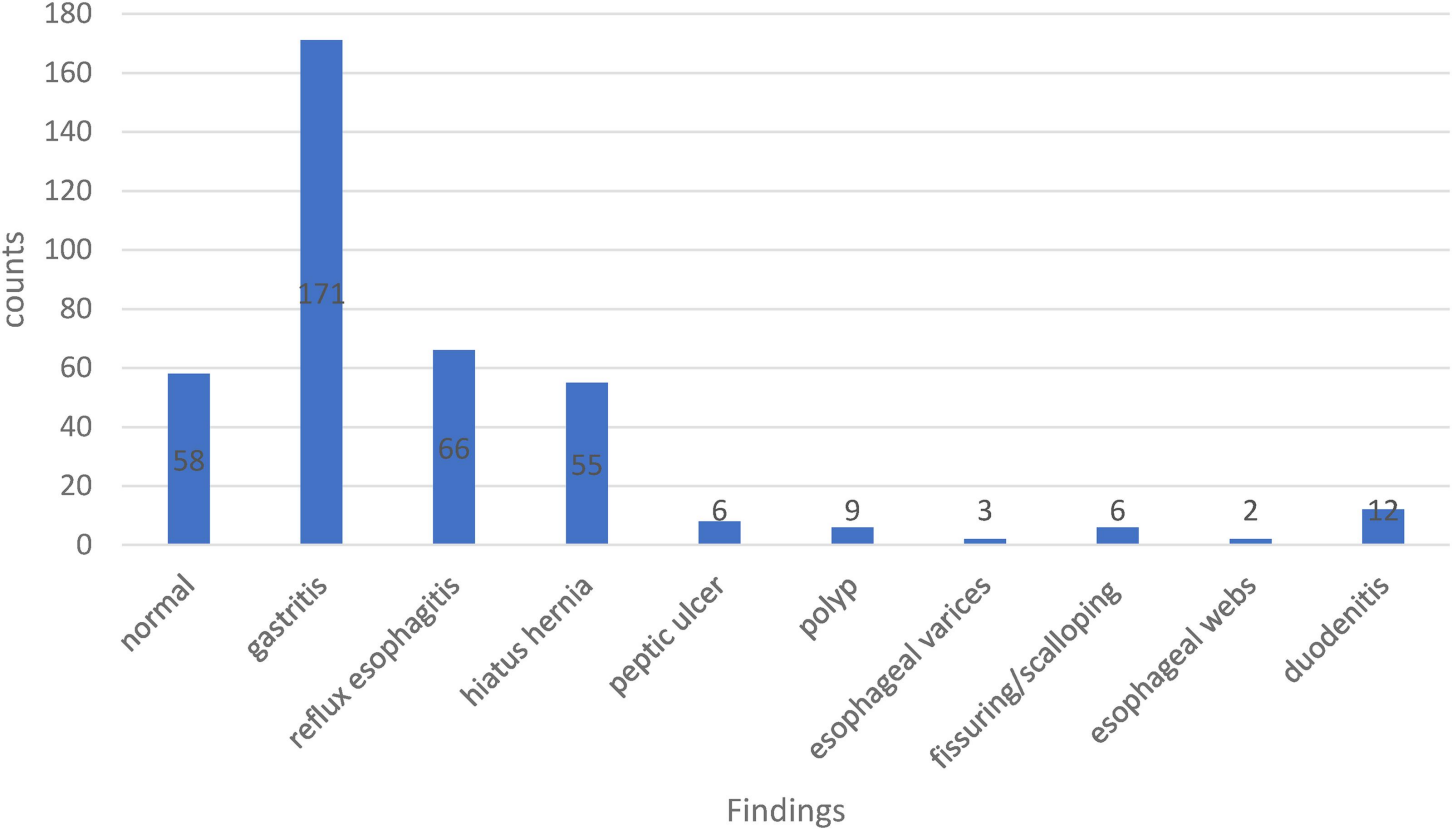
Distribution of upper endoscopic findings in 282 women with iron deficiency anemia. Upper endoscopy results were recorded for women under 50 years of age with iron deficiency anemia at Jordan University Hospital and Al-Hussein Salt Hospital during the period from June 2022 to June 2025. Some patients had more than one abnormal finding.

A total of 282 patients underwent upper endoscopy with duodenal biopsies, and abnormal findings were detected in 224 (79.4%). Gastritis (57.0%) was the most common finding, followed by reflux esophagitis (22.0%) and hiatal hernia (18.3%). Six cases of celiac disease were diagnosed among the cohort (2%). No upper gastrointestinal malignancies were diagnosed Figure 2.

A comparison between patients with abnormal (*n* = 224) and normal (*n* = 58) upper endoscopy findings revealed no significant differences in median age (38.0 vs. 40.0 years, *p* = 0.461), hemoglobin (10.7 vs. 10.6 g/dL, *p* = 0.703), or ferritin levels (6.0 vs. 4.8 ng/mL, *p* = 0.703). The prevalence of symptoms (82.6% vs. 79.3%, *p* = 0.563), history of menorrhagia (41.6% vs. 42.9%, *p* = 0.914), and h. pylori positivity (51.5% vs. 59.0%, *p* = 0.396) were also comparable between the groups.

### Lower endoscopic findings in women under 50 years with iron deficiency anemia

**Figure 3:**
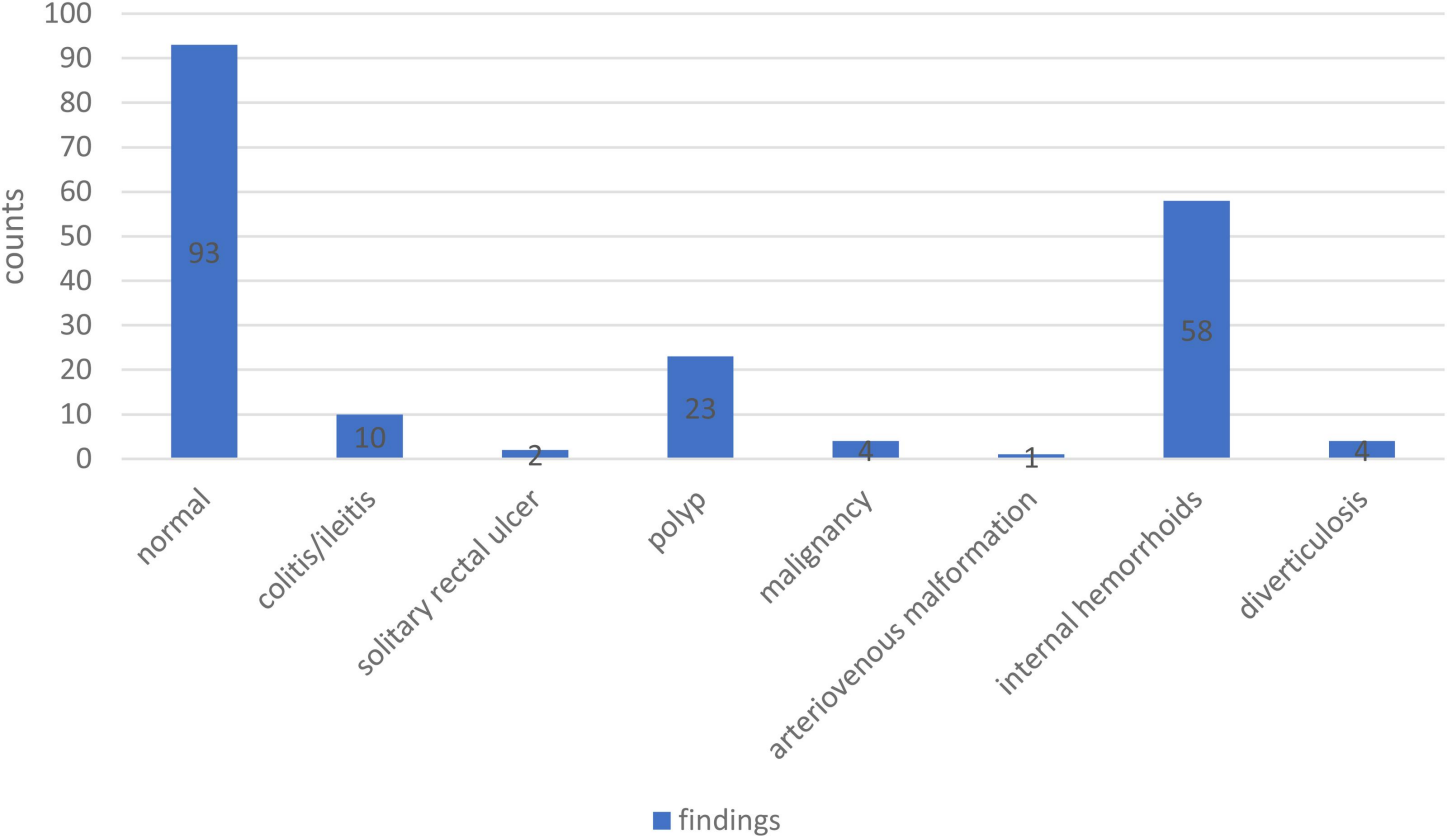
Distribution of lower endoscopic findings in 181 women with iron deficiency anemia. Lower endoscopy results were recorded for women under 50 years of age with iron deficiency anemia at Jordan University Hospital and Al-Hussein Salt Hospital during the period from June 2022 to June 2025. Some patients had more than one abnormal finding.

Lower endoscopy was performed on 181 patients, with abnormal findings detected in 88 (48.6%). The most common finding was internal hemorrhoids, present in 32% of patients who underwent colonoscopy Figure 3. IBD was diagnosed in five (1.7%) patients and CRC in four (1.3%). A comparison between patients with abnormal (*n* = 88) and normal (*n* = 93) colonoscopy findings showed no significant differences in median age (42.0 vs. 39.0 years, *p* = 0.412), Hb level (10.5 vs. 10.7 g/dL, *p* = 0.497), or ferritin level (4.7 vs. 7.0 ng/mL, *p* = 0.116). The prevalence of GI symptoms (86.4% vs. 79.1%, *p* = 0.200), a positive h. pylori result (50.0% vs. 49.3%, *p* = 0.932), and a history of menorrhagia (55.0% vs. 36.0%, *p* = 0.071) were also not significantly different between the groups.

### Analysis of specific diagnosis: celiac disease, IBD and cancer

A subgroup analysis was performed comparing patients with a new diagnosis of celiac disease, IBD, or cancer to the rest of the cohort. These comparisons were exploratory and limited by the small number of cases for each diagnosis. All four patients diagnosed with CRC were symptomatic. However, no statistically significant differences were found between the CRC group and patients without cancer in age (*p* = 0.559), Hb level (*p* = 0.615), ferritin level (*p* = 0.634), overall prevalence of symptoms (*p* = 1.00), history of menorrhagia (*p* = 1.00), or h. pylori status (*p* = 0.213). Similarly, among the six patients diagnosed with celiac disease, no significant associations were found with age (*p* = 0.754), Hb level (*p* = 0.680), ferritin level (*p* = 0.704), the presence of GI symptoms (*p* = 1.00), a history of menorrhagia (*p* = 0.509), or h. pylori status (*p* = 1.00). The five patients diagnosed with IBD were younger than those without IBD (median age 28.0 vs. 39.0 years), a difference that approached, but did not reach, statistical significance (*p* = 0.086). No significant associations were found with other variables.

## Discussion

This study investigated the diagnostic yield of endoscopy in women under 50 years with IDA, focusing on the frequency and location of GI lesions, the prevalence of specific diagnoses like celiac disease, IBD, and malignancy, and the influence of clinical variables on identifying an underlying cause. Overall, the diagnostic yield for a significant endoscopic finding was 19.3%. Upper GI lesions prevailed, with a diagnostic yield of 79.4% for upper endoscopy compared to 48.6% for colonoscopy. The prevalence of serious pathologies was 1.3% for CRC, 2.0% for celiac disease, and 1.7% for IBD. Increasing age was the only variable significantly associated with the presence of a significant endoscopic finding. None of the variables were significantly associated with any of the specific diagnoses. These findings support the evidence that IDA in premenopausal women is multifactorial, with the GI tract contributing to iron deficiency through H. pylori-related lesions, malabsorption, or occult blood loss. Given that the average age of menopause in Jordan was reported as 49.5 years (10) and our cohort had a median age of 39.0 years with the upper range at 49 years, it is reasonable to presume that the majority of our study population was premenopausal.

We found that upper GI lesions, particularly gastritis, were highly prevalent which is consistent with global data identifying the upper GI tract as a major source of IDA. However, the yield of endoscopy varies considerably in the literature. Our findings align with studies such as Vannella et al., who observed gastrointestinal causes in 69% of premenopausal women with IDA, mostly related to malabsorption (11). Annibale et al. also reported high yield of upper GI source in 75% of their cohort, which included older women (12). In contrast, much lower yields have been reported by Carter et al. who found clinically significant gut lesions in only 30% of their cohort, with H. pylori gastritis and celiac disease being the leading etiologies (13).

More than half of those with documented testing for H. pylori among our cohort were positive for the infection. Our prevalence of positive H. pylori cases was higher than the rates reported in Western populations (14) but aligns with the broad range (21%–100%) documented across the Middle East and North Africa region (15). This supports a significant contributing role for H. pylori infection in the development of IDA, which could be mediated through chronic atrophic gastritis and occult blood loss (16).

Looking at rates of specific diagnosis, our reported 2% prevalence for celiac disease is consistent with the 3.2% pooled prevalence reported in a meta-analysis by Mahadev et al. (17), highlighting its contribution to the pathogenesis of IDA in this population. As for malignancy, our observed prevalence of 1.3% for CRC is slightly lower than the 2.7% and 3% rates reported by Green et al. (18) and Bini et al. (19) respectively, which confirms that cancer remains a non-negligible finding even in younger women with IDA. It is worth mentioning that the latter studies also identified clinical predictors such as the presence of symptoms, Hb <10 g/dL, and a positive fecal occult blood test—associations not observed in our cohort. Due to the descriptive study design and the small number of cases with specific disease diagnosis, our study was not powered to identify clinical predictors. Therefore, the absence of an association with these specific diagnoses may reflect type II error rather than true lack of association.

The overall diagnostic yield for any significant lesion also varied widely across studies. Our yield of 19.3% falls between the much higher yields reported by some, such as Abramowitz et al. (28.6% on upper endoscopy) (20), and the considerably lower yields reported by others. For instance, Park et al. (21) and Robson et al. (22) reported diagnostic yields of 6.5% and 7% for upper endoscopy, respectively, particularly in asymptomatic cohorts. Interestingly, Abramowitz et al. also reported that asymptomatic individuals had a higher diagnostic yield than symptomatic ones, a finding that contrasts with conventional clinical reasoning and our own results (20). Table 2 summarizes the diagnostic yields of other studies compared with ours.

**Table 2:** Comparison of endoscopic yields in women with iron deficiency anemia reported in previous studies.

Study	Year	Population	Upper endoscopy yield	Lower endoscopy yield
Vannella et al. (11)	2008	Premenopausal women	68.5%	-
Annibale et al. (12)	2001	Women aged 23–87 years	74.5%	15.7%
Carter et al. (13)	2008	Premenopausal women	26%	5.2%
Green et al. (18)	2004	Premenopausal women	13%	7.2%
Bini et al. (19)	1998	Premenopausal women	7%	6%
Abramowitz et al. (20)	2024	Men and women aged < 45 years	28.6%	8.3%
Park et al. (21)	2006	Premenopausal women	0.9%	5.6%
Robson et al. (22)	2009	Premenopausal women	7%	3.9%

Overall, our results agree with previous studies reporting that upper GI lesions, mostly gastritis, are common in premenopausal women with IDA, regardless of the presence of GI symptoms, and require endoscopic workup. The observed variation in diagnostic yields across studies is likely due to differences in the definitions of significant lesions, local demographics (i.e, prevalence of h. pylori infection and malignancy), and referral patterns that may prioritize patients with a higher chance of having significant findings. Another important factor to consider is the healthcare setting in which the study was performed. Our study was conducted in two insurance-based hospitals where endoscopic procedures are performed with minimal out-of-pocket cost. This likely lead to a lower threshold to investigate IDA and potentially finding a wider range of pathologies than might be observed in more resource-restricted health systems.

Our study has several important strengths. To our knowledge, it is the first to investigate the endoscopic yield in young women with IDA in Jordan and the Middle East. The sample size of 300 women with confirmed IDA is larger than previous studies and provides robust data for analysis. The dual-center design ensures that the findings are more representative of the Jordanian population. Finally, our definition of a significant endoscopic finding consisted of a broad spectrum of gastrointestinal lesions that could explain a patient's iron deficiency. However, certain limitations should be considered. The retrospective nature of the study carries a risk for documentation bias, as evidenced by the missing data regarding H. pylori status and menstrual history. Data on other micronutrients, such as vitamin B12, and markers of inflammation, including C-reactive protein and fecal calprotectin, were frequently missing and thus could not be evaluated as potential predictors of significant endoscopic findings or specific diagnoses. Selection bias is another limitation. Asymptomatic young women with IDA may not seek medical attention, and even when they do, clinicians may prioritize symptomatic patients for endoscopic referral. Consequently, our findings are more generalizable to young women with IDA who have GI symptoms or other clinical features prompting evaluation. Future prospective studies focusing on asymptomatic iron deficient young women are warranted to determine the true prevalence of organic GI lesions in this population. Furthermore, not all participants underwent bidirectional endoscopy; a third of the population did not have a colonoscopy. This incomplete assessment increases the possibility of missed colonic pathology, potentially underestimating the burden of lower GI disease. The small number of cases for specific diagnoses like celiac and cancer limited our statistical power to identify their predictors. Finally, the definition of a “significant finding,” while necessary for analysis, inevitably involves a degree of clinical judgment.

## Conclusion

Our study confirms an important role for GI pathology as a cause of IDA in young Jordanian women, with endoscopic evaluation identifying a potential cause in nearly a fifth of the population. The upper GI tract was the most common site of abnormality, with a high prevalence of h. pylori infection. The prevalence of CRC was low but not negligible. Given the lack of reliable clinical predictors for significant findings, apart from older age, a proactive endoscopic approach is justified to identify underlying gastrointestinal pathology.

## Data Availability

The raw data supporting the conclusions of this article will be made available by the authors, without undue reservation.
